# Stromal categorization in early oral tongue cancer

**DOI:** 10.1007/s00428-020-02930-5

**Published:** 2020-09-21

**Authors:** Alhadi Almangush, Ibrahim O. Bello, Ilkka Heikkinen, Jaana Hagström, Caj Haglund, Luiz Paulo Kowalski, Pentti Nieminen, Ricardo D. Coletta, Antti A. Mäkitie, Tuula Salo, Ilmo Leivo

**Affiliations:** 1 Department of Pathology, University of Helsinki, Haartmaninkatu 3, P.O. Box 21, FIN-00014 Helsinki, Finland; 2 Research Program in Systems Oncology, Faculty of Medicine, University of Helsinki, Helsinki, Finland; 3 Department of Oral and Maxillofacial Diseases, University of Helsinki, Helsinki, Finland; 4 Institute of Biomedicine, Pathology, University of Turku, Turku, Finland; 5 Faculty of Dentistry, University of Misurata, Misurata, Libya; 6 Department of Oral Medicine and Diagnostic Sciences, King Saud University College of Dentistry, Riyadh, Saudi Arabia; 7 Research Programs Unit, Translational Cancer Medicine, University of Helsinki, Helsinki, Finland; 8 Department of Surgery, University of Helsinki and Helsinki University Hospital, Helsinki, Finland; 9 Department of Head and Neck Surgery and Otorhinolaryngology, A.C. Camargo Cancer Center, São Paulo, SP Brazil; 10 Department of Head and Neck Surgery, University of Sao Paulo Medical School, São Paulo, SP Brazil; 11 Medical Informatics and Data Analysis Research Group, University of Oulu, Oulu, Finland; 12 Department of Oral Diagnosis, School of Dentistry, University of Campinas, Piracicaba, São Paulo Brazil; 13 Department of Otorhinolaryngology – Head and Neck Surgery, University of Helsinki and Helsinki University Hospital, Helsinki, Finland; 14 Division of Ear, Nose and Throat Diseases, Department of Clinical Sciences, Intervention and Technology, Karolinska Institutet and Karolinska University Hospital, Stockholm, Sweden; 15 Cancer and Translational Medicine Research Unit, Medical Research Center Oulu, University of Oulu and Oulu University Hospital, Oulu, Finland; 16 Institute of Biomedicine, Pathology, University of Turku and Turku University Hospital, Turku, Finland

**Keywords:** Tumor microenvironment, Stromal categorization, Desmoplastic reaction, Oral tongue squamous cell carcinoma (OTSCC), Survival, Prognosis

## Abstract

Stromal categorization has been used to classify many epithelial cancer types. We assessed the desmoplastic reaction and compared its significance with other stromal characteristics in early (cT1-2N0) oral tongue squamous cell carcinoma (OTSCC). In this multi-institutional study, we included 308 cases treated for early OTSCC at five Finnish university hospitals or at the A.C. Camargo Cancer Center in São Paulo, Brazil. The desmoplastic reaction was classified as immature, intermediate, or mature based on the amount of hyalinized keloid-like collagen and myxoid stroma. We compared the prognostic value of the desmoplastic reaction with a stromal grading system based on tumor-stroma ratio and stromal tumor-infiltrating lymphocytes. We found that a high amount of stroma with a weak infiltration of lymphocytes was associated statistically significantly with a worse disease-free survival with a hazard ratio (HR) of 2.68 (95% CI 1.26–5.69), worse overall survival (HR 2.95, 95% CI 1.69–5.15), and poor disease-specific survival (HR 2.66, 95% CI 1.11–6.33). Tumors having a high amount of stroma with a weak infiltration of lymphocytes were also significantly associated with a high rate of local recurrence (HR 4.13, 95% CI 1.67–10.24), but no significant association was found with lymph node metastasis (HR 1.27, 95% CI 0.37–4.35). Categorization of the stroma based on desmoplastic reaction (immature, intermediate, mature) showed a low prognostic value for early OTSCC in all survival analyses (*P* > 0.05). In conclusion, categorization of the stroma based on the amount of stroma and its infiltrating lymphocytes shows clinical relevance in early OTSCC superior to categorization based on the maturity of stroma.

## Introduction

In solid malignant tumors, stromal cells and immune cells are fundamental components of tumor microenvironment. Findings from recent research have shown that tumor stroma influences antitumor immunity; and the crosstalk between stromal cells and immune cells in the tumor microenvironment has been widely discussed [1]. Moreover, tumor microenvironment can influence tumor angiogenesis, therapeutic responses, and treatment resistance [2]. However, factors related to tumor microenvironment are not considered during risk stratification and treatment planning of patients with oral cancer.

Oral tongue squamous cell carcinoma (OTSCC) is the most frequently diagnosed cancer type in the oral cavity and has been recognized as a distinct subsite for mainly two reasons. First, the tongue is the only part of the oral cavity with a rich lymphatic network, and it consists of muscles not encapsulated by the fascia or bone that could restrict the spread of a tumor [3, 4]. Second, OTSCC is associated with a worse prognosis than carcinomas in other locations of the oral cavity [5]. Moreover, OTSCC is distinct from carcinomas in the base of the tongue where high-risk human papillomavirus (HPV) is involved in pathogenesis. Interestingly, the prognostic value of histopathologic features of SCC varies among the different subsites of the oral cavity [6]. Thus, assessment of prognostic markers in a homogenous cohort of a specific subsite constitutes an epidemiological advantage. Early-stage OTSCC is expected to have a better survival than the advanced stages. Therefore, management of early-stage OTSCC usually consists of surgical resection of the tumor, while multimodality treatments are needed for the advanced stages. However, due to a high rate of recurrences and high cancer-related mortality in early OTSCC [7, 8], multimodality treatment will be necessary in some cases with aggressive behavior. Thus, a prognostic indicator such as stromal categorization is of a high clinical importance to aid in the identification of early-stage OTSCC cases that should receive multimodality treatments.

To date, histopathologic grading of OTSCC is entirely based on tumor characteristics, while stromal characteristics have not been considered in daily pathology practice. Stromal cells and other components of tumor microenvironment have proved to be important in invasion and metastasis of many cancers [9]. Moreover, recent research has emphasized the significance of stromal cells in the progression and prognosis of epithelial tumors [10]. Therefore, assessing both the tumor and its surrounding stroma simultaneously will allow for a better insight into the biological behavior than looking at tumor cells alone.

The desmoplastic reaction has been recognized as an important stromal characteristic in some solid tumors including colorectal cancer, colorectal liver metastases, and pancreatic ductal adenocarcinoma [11–13]. Regarding the desmoplastic reaction, the stroma has been categorized into mature, intermediate, and immature based on morphology and arrangement of collagen and the myxoid nature of stroma. [14]. Of note, the clinical significance of the desmoplastic reaction has not been studied in early OTSCC. Our recent research has shown that the assessment of stromal tumor-infiltrating lymphocytes (TILs) has clinical significance in early OTSCC [15]. In addition, we have evaluated the significance of tumor-stroma ratio (stroma-rich vs. stroma-poor) in early OTSCC [16]. In the present study, we sought to evaluate the prognostic significance of stromal categorization in a large multicenter cohort of early (cT1-2 N0) OTSCC. We report a new model of stromal categorization based on stromal TILs and tumor-stroma ratio. Our aim is to identify the clinically most relevant criteria for a histopathologic classification of tumor microenvironment in early OTSCC that can be observed in routine hematoxylin and eosin (HE)-stained slides.

## Material and methods

A total of 308 cases treated for early OTSCC (cT1-2N0M0) at five Finnish university hospitals or at A.C. Camargo Cancer Center in São Paulo, Brazil, were analyzed in this study. Ethical approval for this study was obtained from the Finnish National Supervisory Authority for Welfare and Health, the ethics committees of each university hospital included, and from the Brazilian Human Research Ethics Committee. The basic clinicopathologic information including patient’s age, gender, stage, and the WHO histopathological grading were retrieved from pathology reports.

HE-stained slides of resection specimens of early OTSCC were used for the assessment of all stromal features in this study. We categorized desmoplastic reaction based on the maturity of stroma as recently described [11, 14] into three groups:

- Mature stroma: Refers to fine mature collagen that is stratified into multiple layers and does not contain any myxoid stroma or keloid-like collagen.
- Intermediate stroma: Refers to stroma that contains keloid-like collagen which is intermingled with mature stroma. There is no myxoid change in this category.
- Immature stroma: Refers to fibrotic stroma that contains myxoid change as determined in a microscopic field of × 40 objective.

All categorization was assessed at the invasive front region, and areas of necrosis were excluded. To recognize the most suitable categorization, we also divided the tumors into two groups as with immature vs. mature stroma as described [13, 17].

**Fig. 1:**
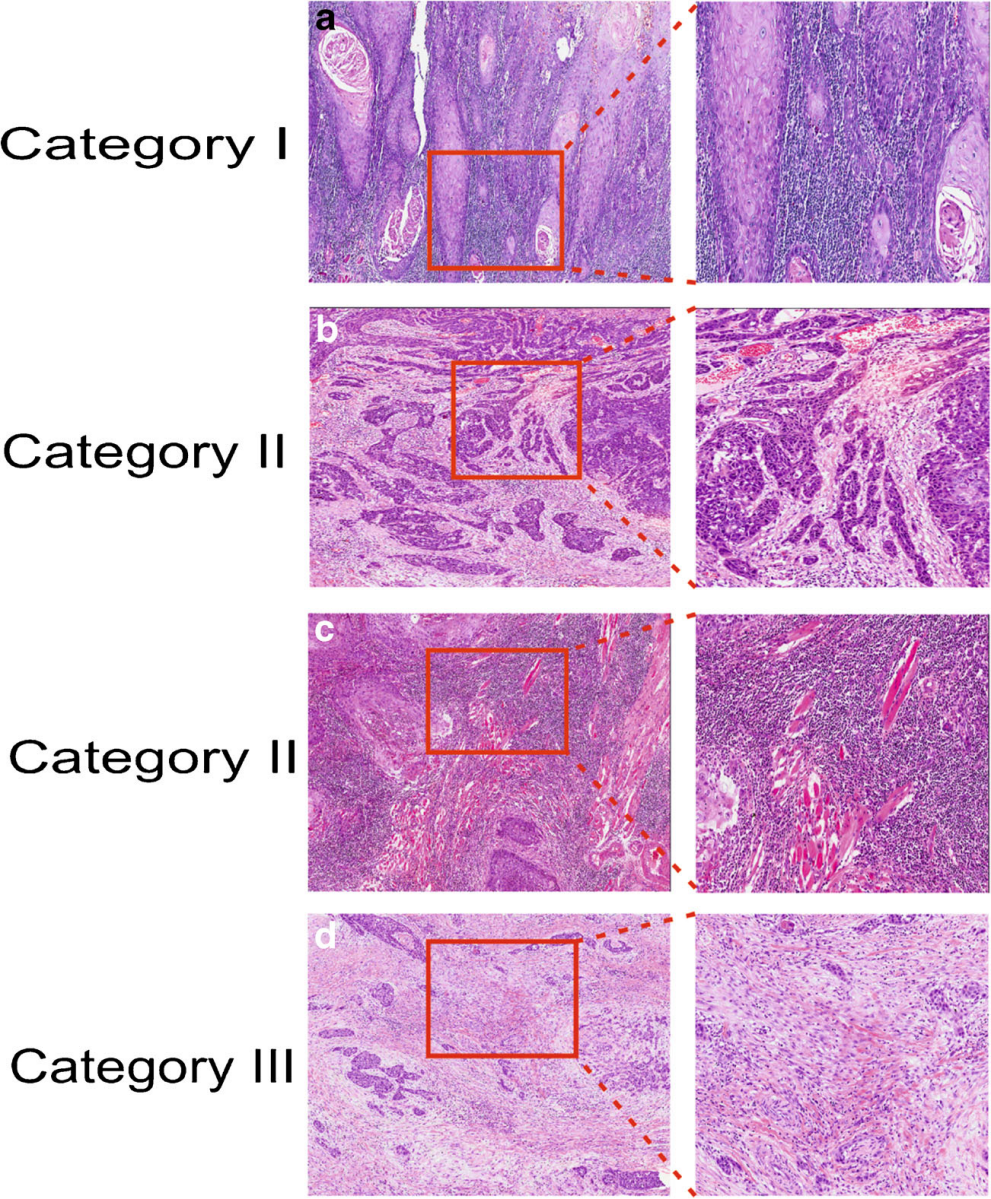
Stromal categorization based on abundance of stroma and infiltrating lymphocytes: (**a**) stromal category I: tumor with stroma-poor pattern (< 50%) and high infiltration of TILs (≥ 20%). (**b**) Stromal category II: tumor with stroma-poor pattern (< 50%) and low infiltration of TILs (< 20%). (**c**) Another example of stromal category II: stroma-rich (≥ 50%) and with high infiltration of TILs (≥ 20%). (**d**) Stromal category III: stroma-rich (≥ 50%) and with scarce infiltration of TILs (< 20%). (**a**) to (**d**) in small magnification (× 4). Inserts show details in higher magnification (× 10)

Furthermore, we proposed a new stromal categorization (Fig. 1) based on stroma ratio and stromal TILs as follows:

- Stromal category I: Low amount of stroma that is highly infiltrated with TILs. Tumors in this category are considered low-risk, and they do not have any adverse stromal features.
- Stromal category II: Low amount of stroma with low TILs or high amount of stroma with high TILs. Category II is considered to represent intermediate risk, and it includes tumors which have only one adverse stromal feature. Thus, combinations formed by only one of the adverse stromal features (either high stromal content of ≥ 50% with high TILs of > 20% or low amount of stroma < 50% with low infiltration of TILs of < 20%) are included. The reason for such groupings is to differentiate category II from category I. It is also important to differentiate category II from category III.
- Stromal category III: Tumors with high amount of stroma having no or a little TILs. Tumors in this category are considered to represent high risk and showed two adverse stromal features (i.e., high stromal content of ≥ 50% and low infiltration of TILs of < 20%).

Assessment of tumor-stroma ratio and stromal TILs (and determination of cutoff points) was originated from our previous studies [15, 16] that followed the recently introduced recommendations [18, 19]. In brief, tumors were classified based on the amount of stroma as stroma-poor which consist of < 50% stromal area in the histological section or as a stroma-rich (≥ 50%). In case of a heterogeneous tumor that had stroma-poor and stroma-rich areas, stroma-rich was considered [16, 19]. For assessment of stromal TILs, stromal area occupied by lymphocytes was estimated in percentage (10%, 20%, 30%, etc.) [15]. A cutoff point of 20% were used to divide the tumors as low stromal TILs (< 20%) or high stromal TILs (≥ 20%).

Training sessions for the assessment of all stromal features were arranged and guided by an experienced head and neck pathologist (IL).

### Statistical analysis

We used IBM SPSS Statistics (version 25) and MedCalc (version 18) for survival analysis and to calculate the prognostic significance of stromal characteristics. Disease-free survival (DFS), overall survival (OS), disease-specific survival (DSS), local recurrence, and lymph node metastasis were analyzed with hazard ratio (HR) and 95% confidence interval (CI) reported for each stromal characteristic in univariable and multivariable analyses using Cox regression. A likelihood-ratio test was used to evaluate whether a prognostic variable makes any independent contribution to the survival. The proportional hazards assumptions of Cox regression were met by the data. Kaplan-Meier survival curves were used to describe the DFS, OS, and DSS using the stromal characteristics. We used log-rank test to evaluate the statistical significance between the survival curves.

## Results

The clinicopathologic characteristics of the present cohort are summarized in Table 1. A total of 177 tumors (66.3%) presented with mature stroma, 21 (7.9 %) with intermediate stroma, and 69 (25.8%) had immature stroma. We were not able to identify the desmoplastic reaction (mature, intermediate, or immature) in 41 tumors, which displayed dense stromal TILs that occupied the areas of interest and made it impossible to recognize the type of underlying stroma. There were 104 (33.8%) well-differentiated, 129 (41.9%) moderately differentiated, and 75 (24.3%) poorly differentiated tumors. Regarding the features of tumor invasion such as worst pattern of invasion [7], there were 75 (24.4%) tumors with a cohesive pattern of invasion (including pushing borders, finger-like growth, and large islands of > 15 cells) and 233 (75.6%) tumors with dissociative pattern of invasion (including small islands ≤ 15 cells and/or tumor satellites). For depth of invasion, 113 (36.7%) tumors were superficial as their depth was < 4 mm, while 195 (63.3%) tumors were considered deep with a depth of invasion of 4 mm or more. The significance and discussion about the worst pattern of invasion and the depth of invasion in early OTSCC were reported in our previous study [7]. There was a significant association between our proposed higher stromal categorization and both dissociative pattern of invasion (*P* < 0.001) and deeper invasion (*P* = 0.029). On the other hand, no significant association (*P* > 0.05) was noted between tumor grade and any of the stromal features including the desmoplastic reaction and our proposed categorization.

**Table 1:** Univariable and multivariable survival analyses according to the traditional clinicopathologic variables and stromal characteristics for early-stage oral tongue squamous cell carcinoma

Univariable analysis							
Variable	No (%)	DFS	*P* value	OS	*P* value	DSS	*P* value
		HR (95% CI)		HR (95% CI)		HR (95% CI)	
Age (years) median: 63			*P* = 0.006		*P* < 0.001		*P* = 0.015
≤ 63	155 (50.3)	1		1		1	
> 63	153 (49.7)	1.84 (1.19–2.83)		2.22 (1.61–3.08)		1.88 (1.13–3.13)	
Gender			*P* = 0.627		*P* = 0.144		*P* = 0.447
Male	163 (52.9)	1		1		1	
Female	145 (47.1)	1.11 (0.73–1.69)		0.79 (0.58–1.08)		1.21 (0.74–1.99)	
Stage			*P* = 0.574		*P* = 0.191		*P* = 0.174
T1N0M0	122 (39.6)	1		1		1	
T2N0M0	186 (60.4)	0.89 (0.58–1.36)		1.25 (0.89–1.75)		1.45 (0.85–2.49)	
WHO histopathologic grading			*P* = 0.741		*P* = 0.208		*P* = 0.203
Well-differentiated	104 (33.8)	1		1		1	
Moderately differentiated	129 (41.9)	1.09 (0.67–1.79)		1.37 (0.96–1.97)		1.71 (0.94–3.14)	
Poorly differentiated	75 (24.3)	1.24 (0.72–2.16)		1.10 (0.72–1.69)		1.58 (0.79–3.16)	
Perineural invasion			*P* = 0.278		*P* = 0.210		*P* = 0.432
None	267 (86.7)	1		1		1	
Present	41 (13.3)	1.37 (0.78–2.43)		1.31 (0.86–1.99)		1.31 (0.67–2.58)	
Desmoplastic reaction*			*P* = 0.706		*P* = 0.621		*P* = 0.278
Mature	177 (66.3)	1		1		1	
Intermediate	21 (7.9)	0.77 (0.31–1.93)		0.97 (0.54–1.74)		0.39 (0.09–1.63)	
Immature	69 (25.8)	1.14 (0.69–1.91)		1.19 (0.82–1.73)		1.25 (0.71–2.19)	
Desmoplastic reaction*			*P* = 0.857		*P* = 0.477		*P* = 0.920
Mature	177 (66.3)	1		1		1	
Immature	90 (33.7)	1.05 (0.65–1.68)		1.13 (0.81–1.59)		1.03 (0.59–1.77)	
Stromal TILs			*P* = 0.011		*P* < 0.001		*P* = 0.021
High (≥ 20%)	257 (83.4)	1		1		1	
Low (< 20%)	51 (16.6)	1.93 (1.16–3.22)		2.46 (1.70–3.57)		2.02 (1.11–3.66)	
Tumor-stroma ratio			*P* = 0.018		*P* = 0.03		*P* = 0.047
Stroma-poor (< 50%)	219 (71.1)	1		1		1	
Stroma-rich (≥ 50%)	89 (28.9)	1.69 (1.09–2.59)		1.41 (1.02–1.96)		1.67 (1.01–2.76)	
Stromal categorization			*P* = 0.006		*P* < 0.001		*P* = 0.023
Category I	191 (62.0)	1		1		1	
Category II	94 (30.5)	1.93 (1.23–3.01)		1.88 (1.35–2.64)		1.89 (1.12–3.21)	
Category III	23 (7.5)	2.24 (1.09–4.59)		2.12 (1.26–3.58)		2.34 (1.03–5.31)	
Stromal categorization			*P* = 0.001		*P* < 0.001		*P* = 0.007
Category I	191 (62.0)	1		1		1	
Category II and III	117 (38.0)	1.98 (1.30–3.02)		1.93 (1.41–2.64)		1.97 (1.20–3.24)	
Multivariable analysis**							
Stromal categorization			*P* = 0.003		*P* < 0.001		*P* = 0.022
Category I	191 (62.0)	1		1		1	
Category II	94 (30.5)	1.94 (1.24–3.03)		1.85 (1.32–2.59)		1.82 (1.07–3.09)	
Category III	23 (7.5)	2.68 (1.26–5.69)		2.95 (1.69–5.15)		2.66 (1.11–6.33)	
Stromal categorization			*P* = 0.001		*P* < 0.001		*P* = 0.009
Category I	191 (62.0)	1		1		1	
Category II and III	117 (38.0)	2.04 (1.34–3.12)		2.01 (1.46–2.76)		1.94 (1.18–3.19)	

*Desmoplastic reaction was assessed in 267 tumors

**Adjusted for age, gender, stage, WHO grade, and perineural invasion

*OS* overall survival, *DSS* disease-specific survival, *DFS* disease-free survival. The statistical significances (*P* values) of variables were assessed using likelihood-ratio test. Note: Stromal TILs and tumor-stroma ratio were analyzed in our previous studies (references 15 and 16), and their prognostic values were compared in this study with desmoplastic reaction and our proposed stromal categorization

The survival analyses (Table 1) revealed no evidence of prognostic significance (*P* > 0.05) for desmoplastic reaction in predicting DFS (HR 1.14, 95% CI 0.69–1.91), OS (HR 1.19, 95% CI 0.82–1.73), or DSS (HR 1.25, 95% CI 0.71–2.19). This was noted also when we classified the tumors into two groups as immature vs. mature (Table 1). In addition, the desmoplastic reaction did not show statistically significant association with local recurrence (HR 0.89, 95% CI 0.42–1.89) or with lymph node metastasis (HR 1.37, 95% CI 0.72–2.61).

**Fig. 2:**
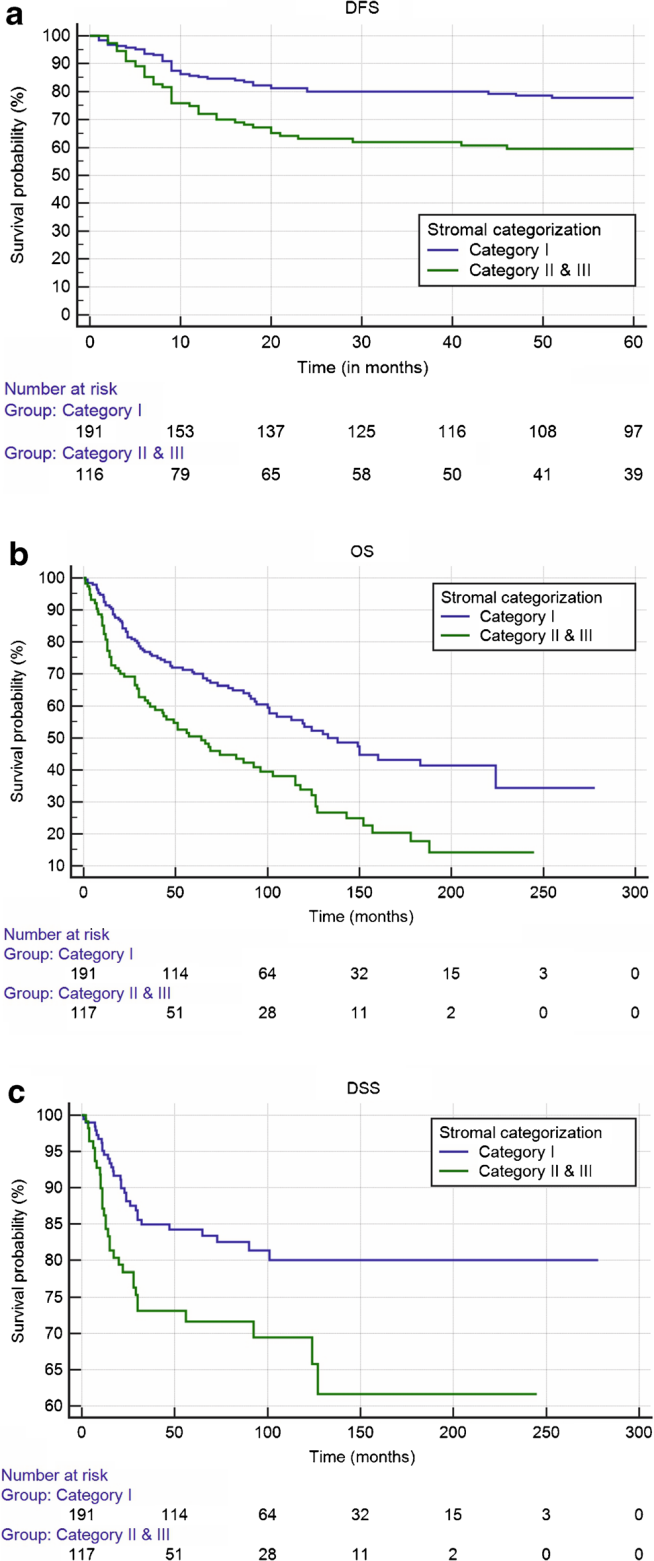
Kaplan-Meier survival plots for 308 cases grouped by the proposed stromal categorization: (**a**) disease-free survival (*P* = 0.001), (**b**) overall survival (*P* < 0.001), (**c**) disease-specific survival (*P* = 0.006)

Our proposed stromal categorization identified a group of tumors in category I with a low amount of stroma which was highly infiltrated by TILs. In survival analysis, these tumors showed favorable prognosis compared with aggressive tumors expressing adverse stromal characteristics (Table 1; Fig. 2). In univariable analysis (Table 1), those aggressive tumors were statistically significantly associated with poor DFS (HR 2.24, 95% CI 1.09–4.59), OS (HR 2.12, 95% CI 1.26–3.58), and worse DSS (HR 2.34, 95% CI 1.03–5.31). These statistically significant associations were confirmed in the multivariable analysis for DFS (HR 2.68, 95% CI 1.26–5.69), OS (HR 2.95, 95% CI 1.69–5.15), and DSS (HR 2.66, 95% CI 1.11–6.33). In addition, such aggressive tumors were significantly associated with a higher rate of local recurrence in univariable (HR 3.64, 95% CI 1.55–8.58) and multivariable (HR 4.13, 95% CI 1.67–10.24) analyses. However, they were not statistically significantly associated with lymph node metastasis either in univariable (HR 1.20, 95% CI 0.36–3.95) or multivariable (HR 1.27, 95% CI 0.37–4.35) analyses.

When we divided our proposed stromal categorization into two groups, a very good prognosis was noted again for tumors with a stromal category I of low stroma amount and high infiltration with TILs as noted in the Kaplan-Meier curves for DFS (*P* = 0.001; Fig. 2a), OS (*P* < 0.001; Fig. 2b), and DSS (*P* = 0.006; Fig. 2c) and also confirmed in the multivariable analysis (Table 1). This method of combining categories was applied to show that the smaller number of cases in category III did not influence the results.

## Discussion

The clinical significance of stromal cells and their characteristics has been studied in many cancers [20]. In OTSCC, however, there is a gap between molecular research that has already documented the importance of tumor stroma and pathology practice which has so far not included the evaluation of stromal characteristics. Currently, histopathological grading according to the WHO criteria which are based on tumor differentiation is often routinely reported in daily pathology practice, although many studies have shown that this grading has a low prognostic value for early-stage OTSCC [7, 21–24]. In addition, differences between tumors of the same type are usually assessed by evaluating tumor-related features (e.g., pattern of invasion and depth of invasion). Of note, it is of high clinical relevance to assess also tumor stroma categorizing cases based on their stroma-related characteristics. To the best of our knowledge, this is the first multicenter study of early OTSCC that analyzed and compared the impact of stromal microenvironment from three aspects: maturity of stroma, amount of stroma (i.e., tumor-stroma ratio), and infiltrating lymphocytes within the stroma (i.e., stromal TILs). We found that stromal categorization that was based on the assessment of tumor-stroma ratio and stromal TILs can serve as a robust tool for prognostication of early OTSCC. Importantly, these two stromal characteristics have shown promising prognostic significance in different subsites of the head and neck cancer [15, 25–29]. We noted that the combination score has a prognostic value superior to each of the single parameters separately (Table 1). Importantly, identification of aggressive early OTSCC based on two adverse prognosticators is obviously more warranted than relying on a single prognosticator [30].

Identification of OTSCC patients at high risk of poor prognosis is sometimes challenging. Current guidelines advise to consider traditional factors such as TNM stage, WHO grade, depth of invasion, and perineural invasion. Of note, none of these factors include assessment of the surrounding tumor microenvironment or the immune response. Moreover, recent research has underlined the importance of predictive model/s that take into consideration the tumor-host interactions to identify high-risk cases and to identify cases that can benefit from specific therapy [31]. Indeed, such identification cannot depend on a single prognostic factor [31].

Stromal tissue consists of mesenchymal cells, immune cells, and vascular cells which are embedded in the extracellular matrix. How these stromal elements influence tumor progression has been a point of major interest for many researchers. In general, stromal cells contribute to the different hallmarks of cancer [32]. Thus, a high stromal content in a tumor has been speculated to reflect active interactions between the tumor cells and the surrounding stroma [33]. Such interactions have been implicated in cancer progression and metastasis. For example, cancer-associated fibroblasts, a major component of stromal tissues, are involved in orchestrating tumor angiogenesis and modulating cancer cells for invasion and metastasis [32]. Furthermore, stromal cells may regulate epithelial cell functions by secretion of growth factors [34]. Therefore, targeting the tumor stroma has been proposed as a strategy for the treatment of cancer [34].

Stromal characteristics/biomarkers have received more attention in recent studies on tumor microenvironment [20]. However, stromal-based classification for early OTSCC that can be assessed in the routine pathology report has not yet been introduced. In this study, we found that evaluation of the desmoplastic reaction and categorization of the stroma based on maturity (i.e., as mature, intermediate, or immature) has a low prognostic significance in early OTSCC. Another disadvantage in the evaluation of the desmoplastic reaction is that a high infiltration of TILs in many cases did not allow for identification of the type of stroma based on its maturity. Therefore, we do not propose assessment of the desmoplastic reaction in early OTSCC. On the other hand, the tumor-stroma ratio has been shown to be a simple stromal characteristic that can be assessed in routine HE-stained slides. It shows a promising prognostic power in different epithelial cancers including the subsites of the head and neck [16, 26]. Abundance of the stroma can form a barrier against the infiltration of immune cells and form an immune desert with only minimal infiltration of TILs usually associated with poor prognosis [35]. Immune cells constitute a major component of the tumor microenvironment [36]. An active immune response against tumors (i.e., anticancer response) is generally associated with good prognosis [37]. Such an active response is presented in this study as a dense expression of stromal TILs, and when associated with a low amount of stroma, it was classified as stromal category I that is associated with a good prognosis (Table 1). Interestingly, recent research has taken a step towards a standardized assessment of TILs, and guidelines have been published by the International TILs Working Group [38]. Similarly, recommendations for scoring of tumor stroma ratio have been published recently [19].

Our current study used visual assessment of stromal features. Digital assessment was not performed and should therefore be considered in future research. In addition, our cohort is retrospective in nature, and prospective studies are needed for validating the significance of our proposed stromal categorization. The genetic background of stromal tissues associated with the high-risk category was not analyzed in this study and should also be considered in future studies. Further research also needs to assess the significance of our proposed stromal categorization in other subsites of the oral cavity. With these drawbacks in mind, our novel categorization of tumor microenvironment is based on the combination of simple stromal features (stroma ratio and stromal TIL infiltration) that can be evaluated simply and implemented in daily histopathological diagnostics using conventional HE-stained sections. A subgroup of early OTSCC which are “stroma-rich” and have a “low infiltration of lymphocytes” has an aggressive behavior and is associated with unfavorable prognosis.
